# Supplementary material for: Enhanced Apoptosis and Loss of Cell Viability in Melanoma Cells by Combined Inhibition of ERK and Mcl-1 Is Related to Loss of Mitochondrial Membrane Potential, Caspase Activation and Upregulation of Proapoptotic Bcl-2 Proteins
Source: Int J Mol Sci. 2023 Mar 4;24(5):4961. doi: 10.3390/ijms24054961 (PMC10002974; doi:10.3390/ijms24054961)
Supplement: Supplementary file 1 [file ijms-24-04961-s001.zip › ijms-2199392 - Supplementary figures S1 - S6.pdf]

Journal: IJMS (ISSN 1422-0067)

Manuscript ID: ijms-2199392

Title: Enhanced apoptosis and loss of cell viability in melanoma cells by combined inhibition of ERK and Mcl-1 is related to loss of mitochondrial membrane potential, caspase activation and upregulation of proapoptotic Bcl-2 proteins

Authors: Zhe Peng , Bernhard Gillissen , Antje Richter , Tobias Sinnberg , Max Schlaak , Jürgen Eberle *

Section: Molecular Oncology

Special Issue: Advances in Melanoma Immunotherapy and Molecular Mechanisms of Drug Resistance

Supplementary figures S1 – S6

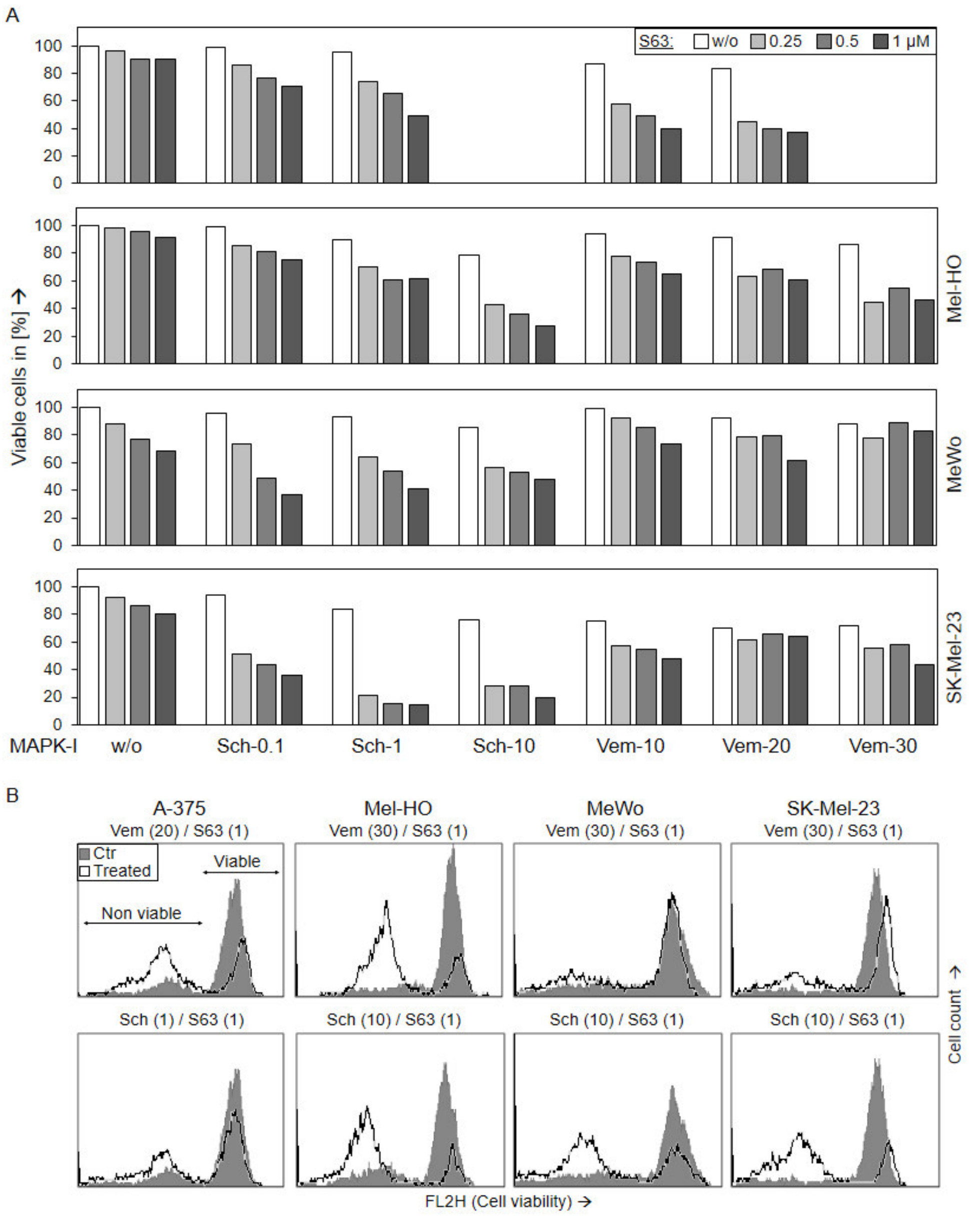

Figure S1. Loss of cell viability (24 h). Melanoma cell lines A-375, Mel-HO, MeWo and SK-Mel-23 were seeded in 24-well plates and were treated with S63845 (S63, 0.25, 0.5, 1.0 µM), SCH772984 (Sch, 0.1, 1.0, 10 µM) and vemurafenib (Vem, 10, 20, 30 µM), as indicated. (**A**) After 24 h, cell viability was determined by calcein-AM staining and flow cytometry. Values represent the percentage of cells with high calcein staining (= viable cells). Effects on cell viability were calculated as percentage of non-treated controls (100%). At least two independent experiments were performed, each one consisting of triplicate values. Median values are shown here. Treatments with 10 µM SCH772984 and 30 µM vemurafenib were not performed in A-375. (**B**) Examples of flow cytometry measurements of cells treated for 24 h with (20/30 µM vemurafenib / 1 µM S63845) or (1/10 µM SCH772984 / 1 µM S63845) are shown as overlays vs. controls (Ctr). Non-viable and viable cell populations are indicated.

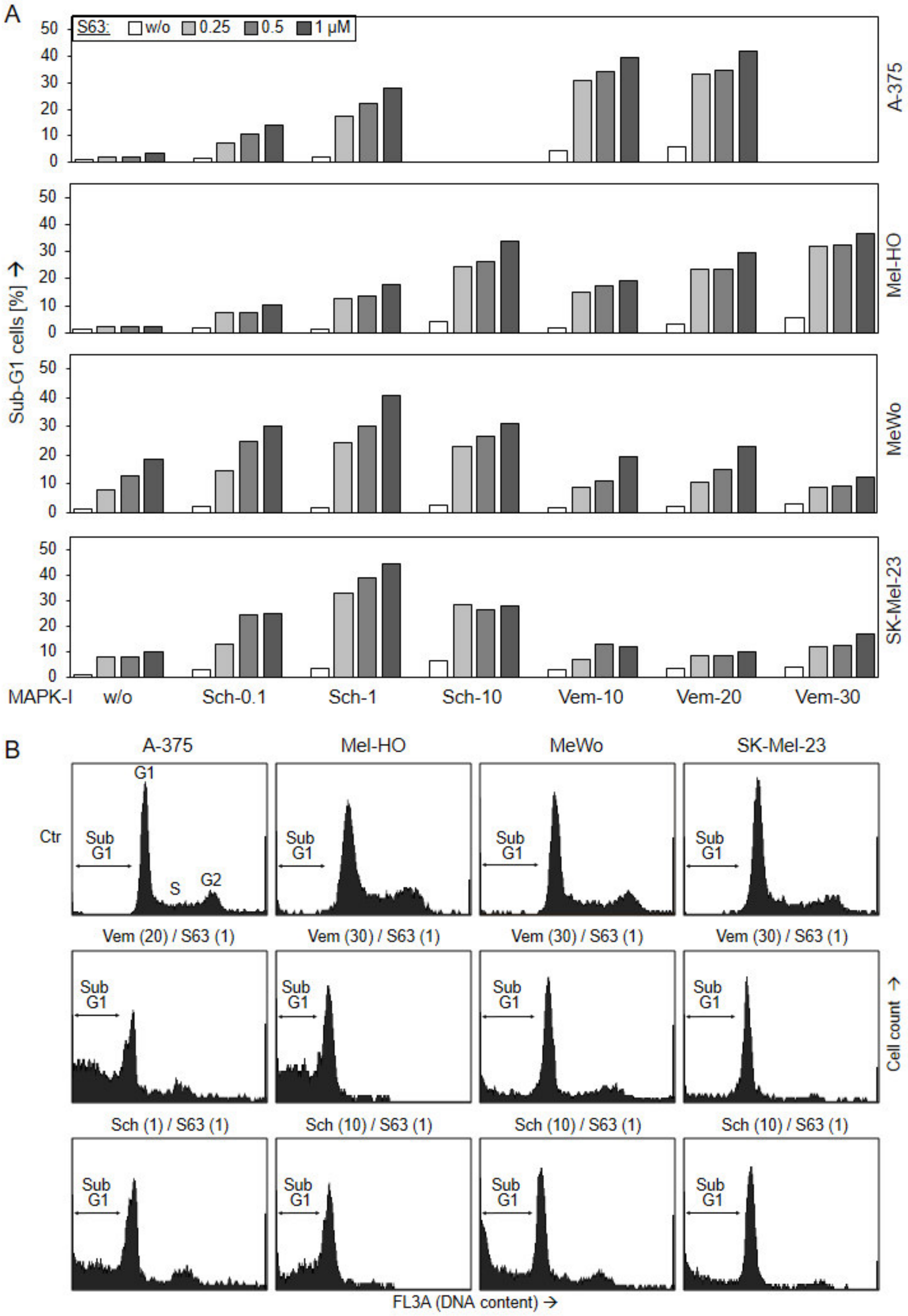

Figure S2. Induction of apoptosis (24 h). Melanoma cell lines A-375, Mel-HO, MeWo and SK-Mel-23 were seeded in 24-well plates and were treated with S63845 (S63, 0.25, 0.5, 1.0 µM), SCH772984 (Sch 0.1, 1.0, 10 µM) and vemurafenib (Vem, 10, 20, 30 µM), as indicated. (A) After 24 h, apoptotic cells were identified by flow cytometry after propidiumiodide staining as sub-G1 cells (cell cycle analyses). At least two independent experiments were performed, each one consisting of triplicate values. Median values are shown here. Treatments with 10 µM SCH772984 and 30 µM vemurafenib were not perfomed in A-375. (B) Examples of flow cytometry measurements of cells treated for 24 h with (20/30 µM vemurafenib / 1 µM S63845) or (1/10 µM SCH772984 / 1 µM S63845) are shown as overlays vs. controls (Ctr). Cell cycle phases G1 (gap 1), S (synthesis), G2 (gap 2) as well as sub-G1 cells are indicated.

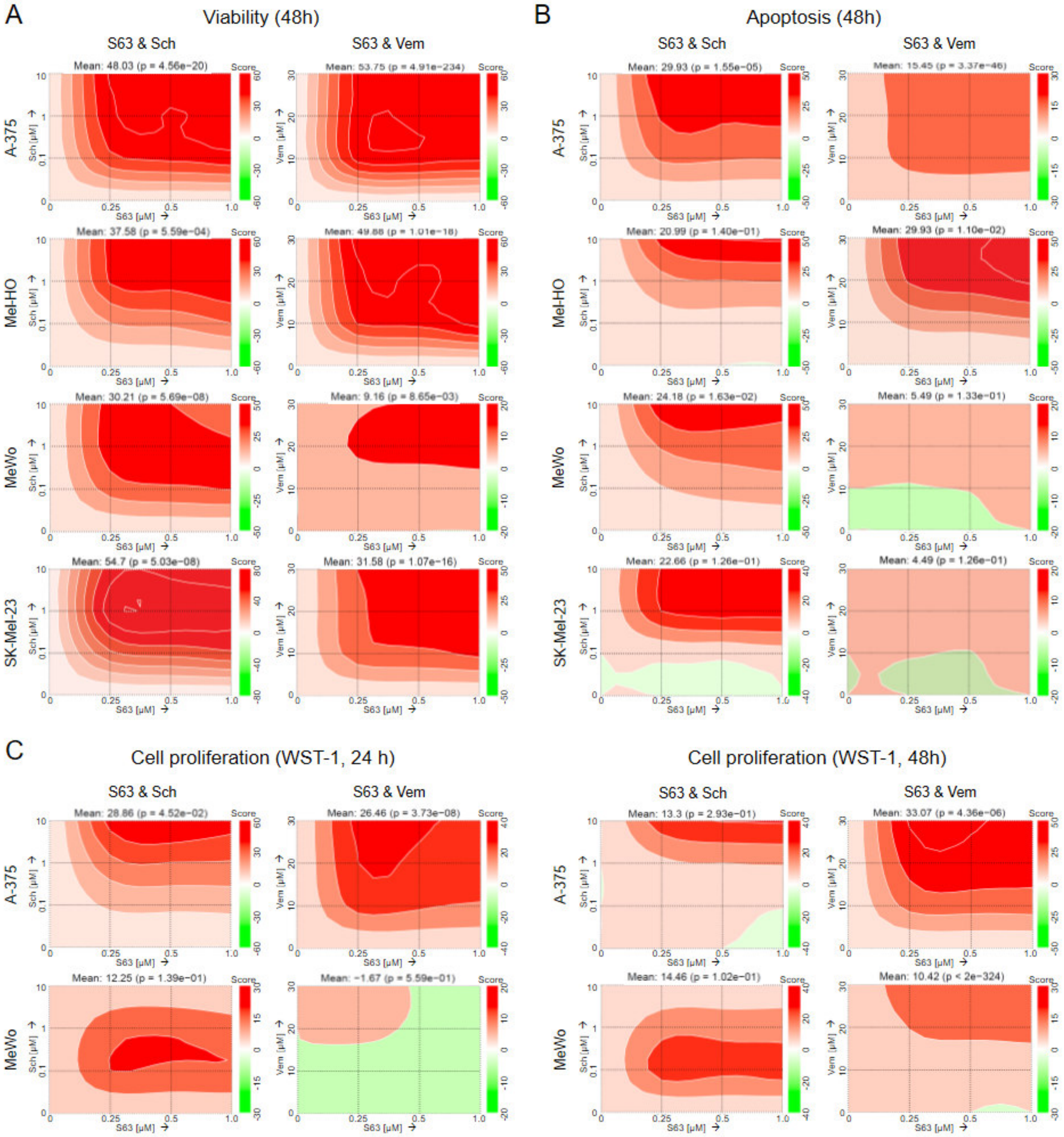

Figure S3. Calculations for synergism. Based on the combination experiments with each three concentrations for the agents SCH772984 (Sch, 0.1, 1.0, 10 µM), vemurafenib (Vem, 10, 20, 30 µM) and S63845 (S63, 0.25, 0.5, 1.0 µM), synergy was calculated in the four melanoma cell lines A-375, Mel-HO, MeWo and SK-Mel-23 by applying the program SyngeryFinder 3.0. Delta scores of > 10 indicate synergism, while scores of ≥ -10 and ≤ 10 indicate additive effects. Calculations were performed for decreased cell viability at 48 h (A), for induced apoptosis at 48 h (B) as well as for decreased cell proliferation at 24 h and 48 h (C).

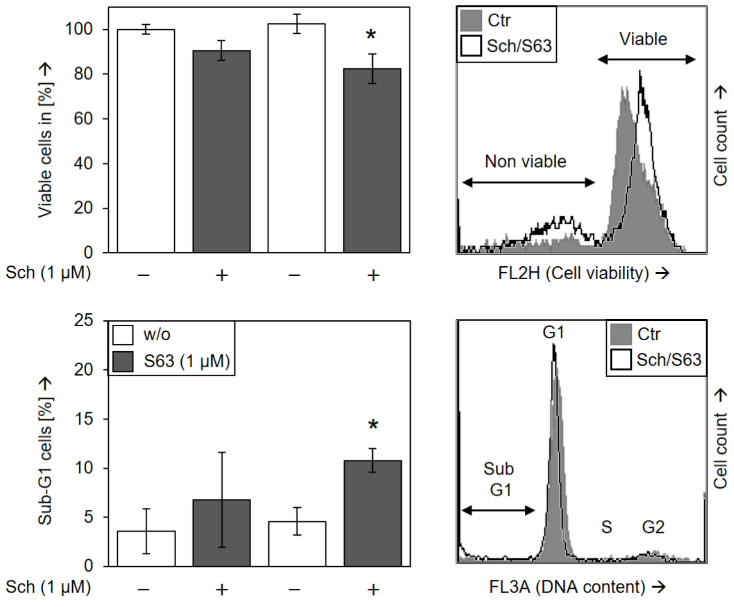

Figure S4. Effects of SCH772984 / S63845 in normal fibroblasts. Three independent cultures of normal human fibroblasts were seeded in 24-well plates and were treated with S63845 (1 µM), SCH772984 (1 µM) or the combination, as indicated. (**Above**) After 48 h, cell viability was determined by calcein-AM staining and flow cytometry (normalized values). (**Below**) Apoptotic cells were identified by flow cytometry after propidium iodide staining as sub-G1 cells (cell cycle analyses,). Each of the three experiments consisted of triplicate values. Mean values and SDs of all individual values (# 9) are shown here. Statistical significance is indicated by asterisks ($p < 0.05$, as compared to control cells). On the right side, examples of flow cytometry readings of combination-treated cells are shown as overlays vs. controls (Ctr). Viable and non-viable cells are indicated as well as cell cycle phases G1 (gap 1), S (synthesis), G2 (gap 2) and sub-G1 cells.

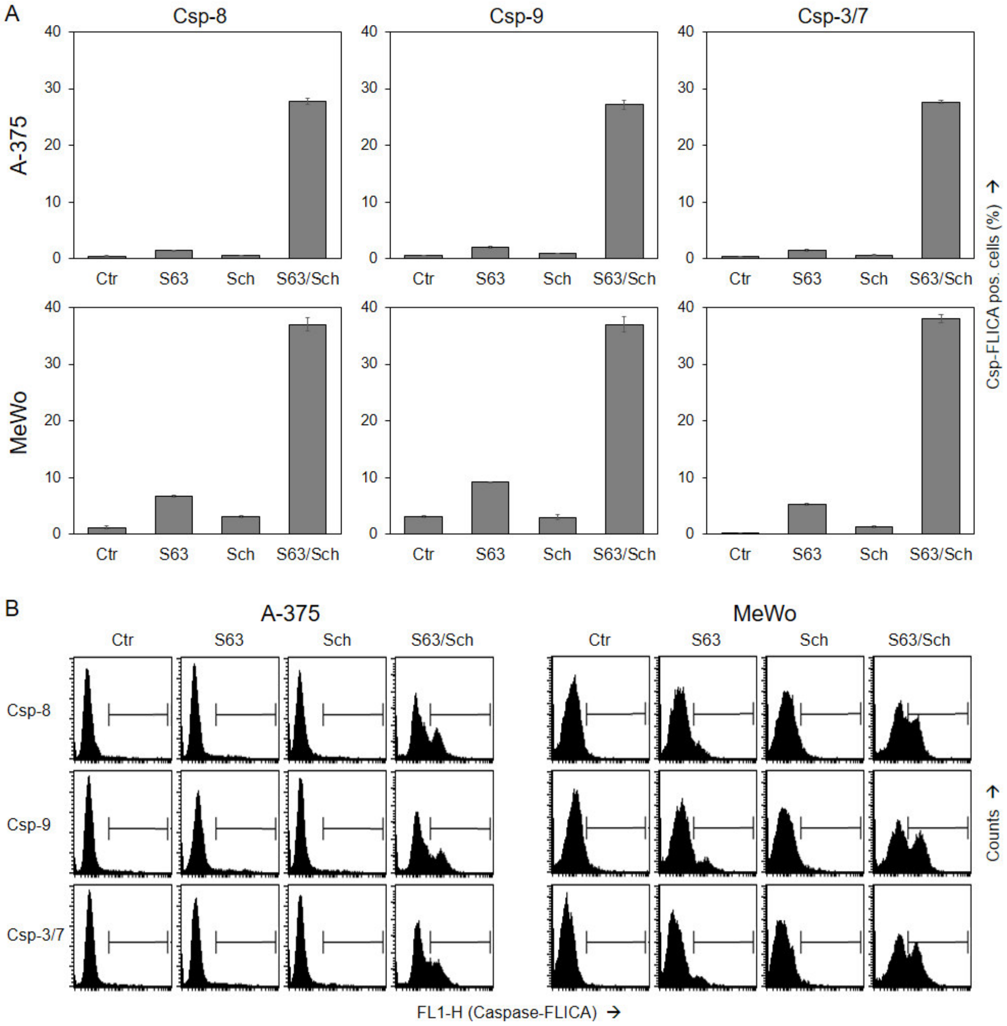

Figure S5. Caspase activation in response to treatment. A-375 and MeWo cells were treated with 1 µM SCH772984 (Sch), 1 µM S63845 (S63) or the combination. At 24 hours after treatment, cells with active caspase-8, caspase-9, or caspase-3/7 were detected by flow cytometry using a FAM-FLICA caspase detection kit. (A) Mean values and SDs were calculated of triplicates; numbers reflect the % of cells with activated caspases. (B) Representative flow cytometry histograms are shown of control and treated cells. Cell fractions with caspase activation are indicated by bars.

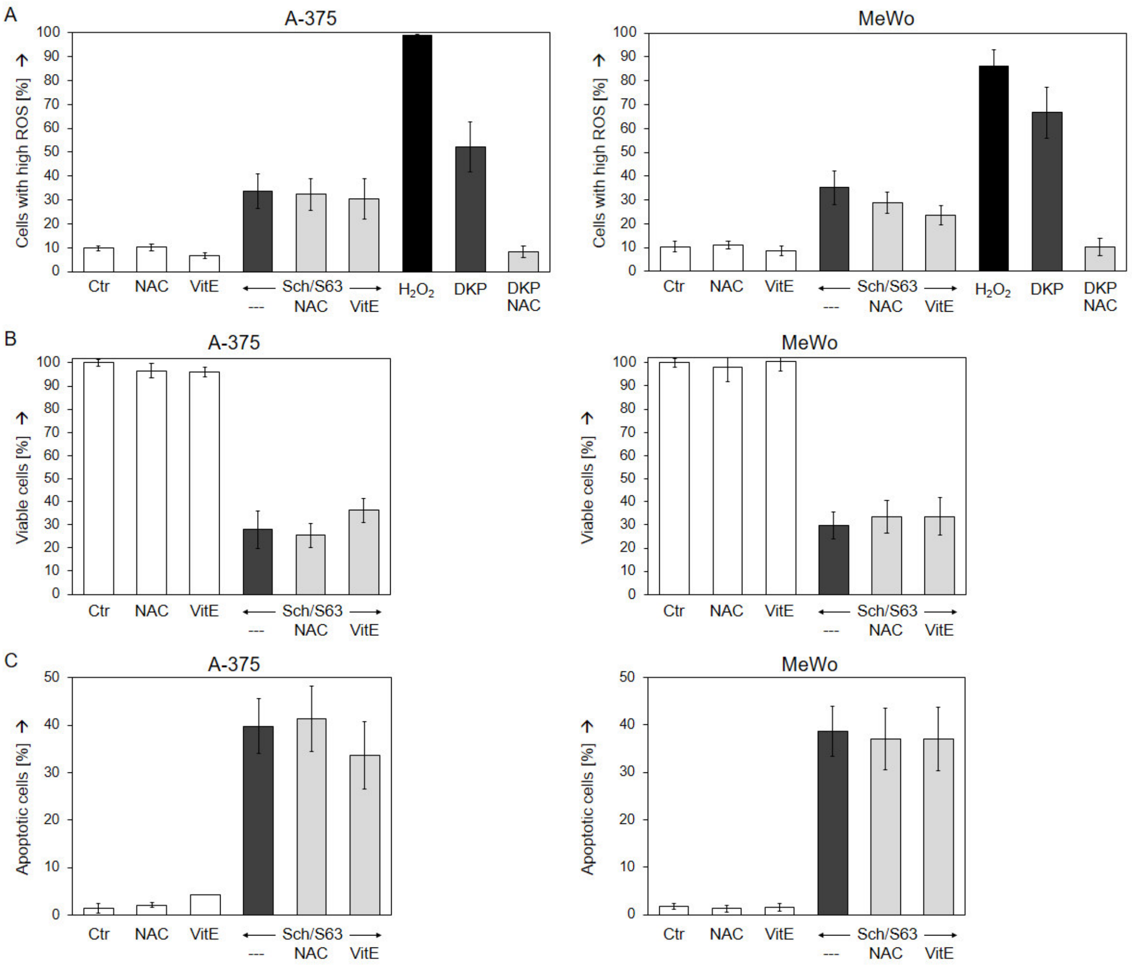

Figure S6. Effects of antioxidants. Melanoma cell lines A-375 and MeWo were treated with the combination 1 µM SCH772984 and 1 µM S63845 (Sch/S63). In addition, cells were pretreated for 1 h with the antioxidants N-acetylcysteine (NAC, 1 mM) or with α-tocopherol (VitE, 1 mM), when indicated. As positive controls, H_2O_2 (1 mM, 1 h) as well an indirubin derivative (DKP-073, 10 µM) were applied. (A) ROS levels were determined by H_2-DCF-DA staining at 24 h. Cell viability (B, calcein staining) and apoptosis (C, sub-G1 analysis) were quantified at 48 h. Mean values and SDs were calculated from three independent experiments, each one based on triplicate values.
